# Nucleated Red Blood Cells Secrete Haptoglobin to Induce Immunosuppressive Function in Monocytes

**DOI:** 10.1155/jimr/8085784

**Published:** 2025-02-20

**Authors:** Shusuke Takeuchi, Satoshi Fujiyama, Motomichi Nagafuji, Miyuki Mayumi, Makoto Saito, Mana Obata-Yasuoka, Hiromi Hamada, Yayoi Miyazono, Hidetoshi Takada

**Affiliations:** ^1^Department of Pediatrics, University of Tsukuba Hospital, Tsukuba, Ibaraki, Japan; ^2^Department of Pediatrics, Ibaraki Prefectural Central Hospital, Kasama, Ibaraki, Japan; ^3^Department of Child Health, Institute of Medicine, University of Tsukuba, Tsukuba, Ibaraki, Japan; ^4^Department of Obstetrics and Gynecology, Institute of Medicine, University of Tsukuba, Tsukuba, Ibaraki, Japan

**Keywords:** haptoglobin, IL-10, monocyte, nucleated red blood cell, umbilical cord blood

## Abstract

Nucleated red blood cells (NRBCs) are precursors of red blood cells (RBCs), but also possess variety of immunomodulatory effects. However, among the three types of NRBCs, the immunological effects of human CD45− NRBCs remain largely unknown. We have previously shown that cord blood-derived CD45− NRBCs and adult peripheral blood-derived monocytes cocultured in a lipopolysaccharide (LPS)-stimulated indirect coculture system that avoided cell-to-cell contact, increase IL-10 and decrease TNF-*α* secretion, suggesting an immunosuppressive function of CD45− NRBCs via an unknown soluble factor. The peripheral blood of fetuses and neonates has abundant NRBCs and is physiologically polycythemic, which may lead to the peripheral accumulation of toxic plasma-free hemoglobin. Plasma-free hemoglobin binds to haptoglobin, forming a haptoglobin–hemoglobin complex, which is processed within monocytes via the CD163− heme oxygenase 1 (HO-1) axis and secretes IL-10. Therefore, we hypothesized that NRBCs secrete haptoglobin and induce the immunosuppressive function of monocytes by activating the CD163−HO-1 axis. We found that immunosuppressive response decreased when the coculture medium was supplemented with an anti-CD163 blocking antibody or the HO-1 inhibitor zinc protoporphyrin IX (ZnPP-IX). Haptoglobin levels in the culture medium containing NRBCs were high and expressed the haptoglobin gene. Thus, CD45− NRBCs secreted haptoglobin and activated the immunosuppressive function of monocytes.

## 1. Introduction

Nucleated red blood cells (NRBCs) are the precursors of red blood cells (RBCs) and are physiologically observed in the peripheral blood of neonates [1]. NRBC counts in peripheral blood increase during systemic inflammation, such as clinical chorioamnionitis and early sepsis [2], and are known to possess various immunomodulatory functions. For instance, NRBCs express the enzyme arginase-2 that suppresses pro-inflammatory cytokine secretion [3], secretes V-domain immunoglobulin suppressor of T cell activation (VISTA) that stimulates immunosuppressive transforming growth factor (TGF)-*β* [4] and produces reactive oxygen species (ROS) that suppress inflammatory cytokine production [5].

NRBCs are classified into three stages: proerythroblasts (pro-EBs), accounting for 5%; basophilic EBs (baso-EBs), accounting for 10%; and polychromatic or orthochromatic EBs (poly/ortho-EBs), accounting for 85% [6]. The expression of cell surface markers of NRBCs varies according to the developmental stage. The expression of CD71, CD117, and CD105 is observed in the most immature stages, but CD117 is lost in baso-EBs, CD105 is lost in poly/ortho-EBs, and CD45 decreases progressively with differentiation [6, 7]. CD45− NRBCs represent approximately 80%–90% of NRBCs [7]. Immunosuppressive effects of CD45+ NRBCs mediated by arginase-2 and ROS have been established [4, 5, 8–10]. However, although CD45− NRBCs express genes relevant to immunomodulation [7], they lack expression of immunoregulatory molecules, such as ROS [5], and their immunomodulatory function remains largely unknown. We have previously reported that CD45− NRBCs exert an immunosuppressive function on adult monocytes without cell-to-cell contact in vitro, for example, monocyte IL-10 and TNF-*α* production levels after the stimulation with lipopolysaccharide (LPS; 100 ng/mL) into 270% and 35% in the presence of CD45− NRBC, respectively, suggesting the involvement of soluble factor underlying the immunosuppressive mechanism [11]. However, the suppressive mechanism has not been identified.

The plasma concentration of haptoglobin, an acute-phase protein synthesized in the liver, correlates with the severity of inflammatory diseases [12]. Haptoglobin detoxifies plasma-free hemoglobin by forming a hemoglobin–haptoglobin complex, which binds to the group B scavenger receptor cysteine-rich superfamily CD163 expressed on the cell surface of phagocytes [13]. Subsequently, the molecular complex is endocytosed and degraded by heme oxygenase 1 (HO-1) in lysosomes, facilitating the production of the anti-inflammatory cytokine, IL-10 [14]. IL-10 suppresses LPS-induced TNF-*α* production by activating signal transducers and activator of transcription 3 (Stat3) [15]. HO-1 deficiency causes severe hemolysis and chronic inflammation, accompanied by increased levels of plasma-free hemoglobin and haptoglobin and an increased NRBC number in the peripheral blood [16]. Therefore, we hypothesized that NRBCs can secrete haptoglobin, facilitating the elimination of toxic plasma-free hemoglobin and improving iron efficiency in EB islets, thereby, inducing an anti-inflammatory response via the CD163−HO-1 axis in phagocytes.

## 2. Result and Discussion

### 2.1. Activation of Hp-HO-1 Axis Increased IL-10 and Decreased TNF-*α* Production in Monocytes

We have previously reported that monocyte inflammatory cytokine production is suppressed in the presence of umbilical cord blood (CB) NRBCs [11]. Monocyte-derived IL-10 mediated this suppressive function because the anti-IL-10 antibody blocked the suppressive function of NRBC in the coculture system [11]. In this study, we focused on the mechanism of enhanced IL-10 production in the presence of NRBCs using monocytes and NRBCs from the same CB sample to investigate immunoregulatory mechanisms of NRBC in neonate.

**Figure 1:**
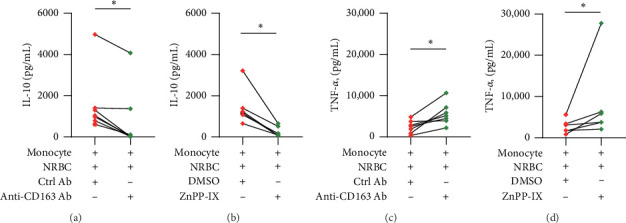
Soluble factor secreted from NRBC induces an anti-inflammatory response in monocytes via activation of the CD163−HO-1 axis. (A) IL-10 concentration (pg/mL) was measured in wells treated with the 10 ng/mL anti-human CD163 mAb (clone RM3/1, anti-CD163 Ab) or the 10 ng/mL control antibody (Ctrl Ab; *n* = 7; Table S1). Median IL-10 concentrations in control: 1007.3 pg/mL and anti CD163 Ab: 82.8 pg/mL. (B) 10 µM ZnPP-IX or dimethyl sulfoxide (DMSO; *n* = 6). Median IL-10 concentrations with DMSO: 1192.1 pg/mL and ZnPP-IX: 133.8 pg/mL. (C) TNF-*α* concentration (pg/mL) was measured in wells treated with the 10 ng/mL anti-CD163 Ab or the 10 ng/mL Ctrl Ab (*n* = 7). Median TNF-*α* concentrations in control: 2238.5 pg/mL and anti CD163 Ab: 4803.3 pg/mL. (D) 10 µM ZnPP-IX or DMSO (*n* = 6). Median TNF-*α* concentrations with DMSO: 2434.7 pg/mL and ZnPP-IX: 4797.1 pg/mL. Monocytes were treated with CD163 Ab, ZnPP-IX, and control reagents 30 min before and during coculture. Each dot that connects with a solid line between the groups represents samples derived from the same individual CB. Each experiment was performed independently. (A–D) Each analyzed cells derived from the same cord blood samples, and one sample was used for both experiments (Table S1). The data were analyzed using the Wilcoxon signed-rank test. *⁣*^*∗*^*p* < 0.05. HO-1, heme oxygenase 1; IL-10, interleukin 10; LPS, lipopolysaccharide; mAb, monoclonal antibody; NRBC, nucleated red blood cell; TNF-*α*, tumor necrosis factor-*α*; ZnPP-IX, zinc protoporphyrin-IX.

To assess whether the CD163−HO-1 axis was involved in the increased production of IL-10 in the presence of NRBCs, we applied the anti-CD163 blocking antibody [14] or isotype IgG control antibody and HO-1 inhibitor zinc protoporphyrin IX (ZnPP-IX) [17] or DSMO carrier control to the coculture medium followed by LPS stimulation and analyzed the concentrations of IL-10 and TNF-*α*. By adding the anti-CD163 blocking antibody or ZnPP-IX, the positive effect of NRBCs on IL-10 production was significantly reduced (*p* < 0.05; Figure 1A,B; *n* = 7 and 6, respectively; Table S1) and TNF-*α* production significantly increased by blocking CD163−HO-1 axis (*p* < 0.05; Figures 1C,1; *n* = 7 and 6, respectively; Table S1) compared to the control sample. This observation suggested that the increase in IL-10 production by coculturing NRBCs and CB-derived monocytes was mediated by the activation of the CD163−HO-1 axis of monocytes.

### 2.2. NRBCs Secreted Haptoglobin to Induce an Anti-Inflammatory Response in Monocytes

**Figure 2:**
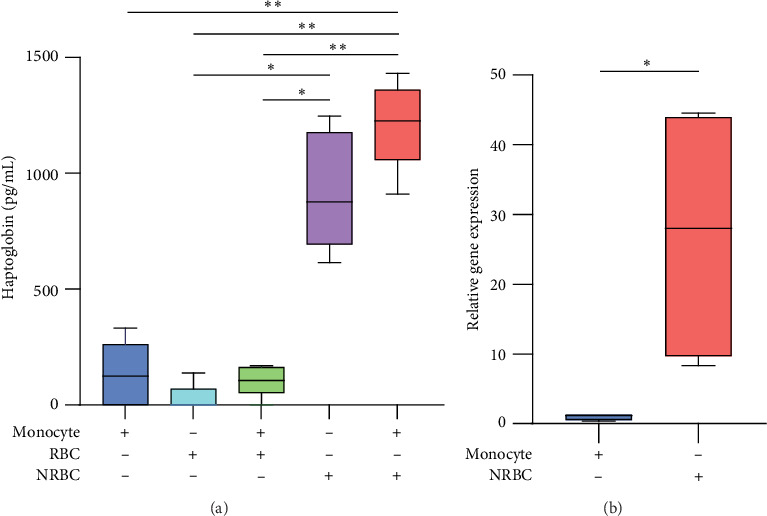
NRBCs secrete haptoglobin to induce an anti-inflammatory response in monocytes. (A) The concentration of haptoglobin (pg/mL) in culture supernatants of monocytes alone or with RBCs or NRBCs was measured using enzyme-linked immunosorbent assay (*n* = 5; Table S1). The median concentrations of haptoglobin in monocyte: 192.1 pg/mL, RBC: 0 pg/mL, monocyte with RBC: 105.8 pg/mL, NRBC: 877.4 pg/mL, and monocyte with NRBC: 1226.6 pg/mL. (B) NRBCs and monocytes were collected separately from the indirect coculture system after coculture. Total RNA was extracted and analyzed for relative gene expression levels of *haptoglobin*. *β*-actin expression was used as the internal control (*n* = 5). The box represents 50% of the values between the 25th and 75th percentiles, and the whiskers represent the range. Each experiment was conducted more than three times. Haptoglobin was analyzed using the Kruskal–Wallis test with Holm correction. Relative gene expression was analyzed using two-sample *t*-tests. *⁣*^*∗∗*^p  < 0.01, *⁣*^*∗*^*p* < 0.05. LPS, lipopolysaccharide; NRBC, nucleated red blood cell; RBC, red blood cell.

Next, we analyzed whether the ligand of the CD163 receptor, the haptoglobin–hemoglobin complex, could mediate the anti-inflammatory function in the LPS-stimulated NRBC-monocyte indirect coculture system. We measured the haptoglobin concentration in the culture medium using an enzyme-linked immunosorbent assay and found significant increase in haptoglobin levels in the NRBC-monocyte coculture medium in the presence of LPS (*p* < 0.01). In contrast to the NRBCs, mature RBCs did not produce haptoglobin (Figure 2A; *n* = 5; Table S1). Furthermore, the mRNA expression of the haptoglobin gene (*HP*) was significantly increased in purified NRBCs after coculture, but not in purified monocytes (*p* < 0.05; Figure 2B; *n* = 5; Table S1). These results suggest that haptoglobin was produced by NRBCs. Collectively, the anti-inflammatory effect of NRBCs on LPS-stimulated monocytes in the indirect coculture system was mediated by NRBC-derived haptoglobin, which formed a complex with hemoglobin and activated the CD163−HO-1 axis of monocytes.

### 2.3. Data Limitations and Perspectives

In this study, we did not elucidate the factors inducing the production of haptoglobin from NRBCs or regulating its secretion from NRBCs. Haptoglobin is an acute response protein and increases in plasma by inflammatory cytokines such as IL-6 [18]. NRBC count in the peripheral blood increases in the inflammatory state and correlates more with IL-6 stimulation than with hematopoietic stimulation by erythropoietin [19]. These findings suggest that NRBCs actively increase haptoglobin levels during the inflammatory state to inhibit further inflammation, which could cause damage to the host. However, RNA sequencing of bone marrow-derived NRBCs from healthy adults has shown relative activation of the haptoglobin production pathway in CD45− NRBCs [7]. This suggests that NRBCs not only actively suppress inflammation, but produces haptoglobin under a steady physiological state, ensuring that inflammation is not triggered by toxic plasma-free hemoglobin leaking from disabling hematopoiesis that occurs at a certain rate where erythropoiesis is active [20]. Further studies on the regulatory mechanisms and the physiological roles of haptoglobin in NRBCs are warranted.

## 3. Conclusion

We identified CB-derived CD45− NRBCs secrete haptoglobin and exert immune regulatory effects on monocytes by activating the CD163−HO-1 axis. Peripheral NRBC counts correlate with the onset of neonatal sepsis and the severity of the inflammatory disease [2]. Therefore, NRBCs may actively suppress excessive inflammation that is dangerous to the self in systemic inflammatory conditions such as sepsis and systemic juvenile idiopathic arthritis [20] by increasing haptoglobin levels and suppressing inflammatory function of monocytes. Furthermore, NRBCs may inhibit inflammation induced by free hemoglobin in physiologically polycythemic fetuses and neonate that may lead to peripheral accumulation of toxic-free hemoglobin [21]. By enhancing the functional pathway carried out by NRBCs specifically in the body, it may be possible to improve these pathological conditions and excessive autoinflammation. Further studies on the detailed regulatory mechanisms and physiological roles of NRBC-derived haptoglobin are warranted.

## 4. Materials and Methods

### 4.1. Isolation of Monocytes and NRBCs

This study was approved by the Ethics Review Committee of the Tsukuba University Hospital (R02-321). Written informed consent was obtained from all participants. Fresh human CB samples were collected from healthy full-term mothers with uncomplicated pregnancies who underwent elective cesarean section, in cases of previous cesarean section or abnormal position of the fetus. Cord blood samples from mothers undergoing C-sections after onset of labor were excluded to reduce the impact of fetal stress on immune status in cord blood. Cord blood from newborns who required treatment after birth was excluded. The cord blood samples were stored at −4°C until isolation. Total of 22 cord blood samples were collected and used for each experiment (Table S1). CB mononuclear cells (CBMCs) were isolated within 24 h via density gradient centrifugation using Lymphoprep (Lymphoprep, Stemcell Technologies, Vancouver, Canada) and SepMate tubes (SepMate, Stemcell Technologies, Vancouver, Canada). CD45+ CD61+ cells were magnetically isolated from CBMCs using an anti-CD45 monoclonal antibody (mAb) and anti-CD61 mAb conjugated microbeads. RBCs were collected from the lower layers of SepMate tubes. CD45+ CD61+ cells were further stained with fluorescein isothiocyanate (FITC)-conjugated anti-CD14 mAb, peridinin chlorophyll protein-cyanine5.5 (PerCPcy5.5)-conjugated anti-CD16 mAb and propidium iodide (PI) to isolate CD14+ CD16− classical monocytes using flow cytometry (MoFlo XDP, Beckman Coulter, CA, USA). NRBCs were isolated from the remaining CD45− CD61− fraction using FITC-conjugated anti-CD36 mAb and anti-FITC mAb-conjugated microbeads. The purity of the CD14+ CD16− monocytes and NRBCs was more than 95%, confirmed by the flow cytometric analysis (Figure S2A,B, respectively). Microbeads were purchased from Stemcell Technologies. Anti-CD14, anti-CD16, anti-CD36, anti-CD45, and anti-CD235a monoclonal antibodies were purchased from BD Biosciences (Franklin Lakes, NJ, USA). The anti-CD71 monoclonal antibodies was purchased from eBiosciences (San Diego, CA, USA). Flow cytometric analysis was performed using a BD LSR Fortessa X-20 (BD Biosciences).

### 4.2. Cell Coculture and Stimulation

Purified CD14+ CD16− monocytes, RBCs, and NRBCs were resuspended in RPMI-1640 culture medium (Gibco Laboratories, Grand Island, NY, USA) and supplemented with 10% heat-inactivated fetal calf serum (Gibco Laboratories). The interaction of the NRBCs and monocytes was analyzed using a trans-well system with a pore size of 0.4 µm (NUNC, Roskilde, Denmark) without cell-to-cell contact. The CD14+ CD16− monocytes were cultured alone or with 1 × 10^7^ cells/mL NRBCs in 24-well plates at a density of 5 × 10^5^ cells/mL with LPS (Sigma–Aldrich). Culture supernatant was collected 36 h after coculture and stored at −80°C for further subsequent analysis. Monocytes and NRBCs were dissolved in ISOGEN Ⅱ (NIPPON GENE, Tokyo, Japan) and stored at −80°C for RNA extraction. For cell signal blocking analysis, 10 ng/mL anti-human CD163 mAb (RM3/1; Gene Tex, Irvine, CA, USA) or 10 µM ZnPP (Cayman Chemical, Ann Arbor, MI, US) were added to the CD14+ CD16− monocyte culture medium 30 min before coculture.

### 4.3. Cytokine Concentration Analysis

The concentrations of TNF-*α* and IL-10 in the culture supernatant were measured using the cytometric bead array (CBA) Human Inflammatory kit (BD Biosciences) following the manufacturer's instructions. The data were analyzed using the CBA software (Version 4.0, BD Biosciences).

### 4.4. Haptoglobin Concentration Analysis

Haptoglobin levels were measured in the supernatant from each well using DuoSet ELISA kits for haptoglobin (R&D Systems).

### 4.5. Gene Expression Analysis

Semiquantitative polymerase chain reaction (PCR) was performed using the SYBR Green PCR Master Mix (Applied Biosystems, Waltham, MA, USA) following the manufacturer's protocol. Each reaction was performed using the QuantStudio 5 Real-Time PCR System for Human Identification (Applied Biosystems). The expression data for each gene of interest were normalized against *β*-actin expression. The HP primers prepared were 5′-ATGGCTATGTGGAGCACTCG-3′ (forward) and 5′-GAAAGCTGCCTTTGGCATCC-3′ (reverse). *β*-actin primer sequences were 5′-AGAGAGGCATCCTCACCCTG-3′ (forward) and 5′-GATAGCACAGCCTGGATAGCA-3′ (reverse). The data were analyzed using the *ΔΔ*Ct method.

### 4.6. Statistical Analysis

All data were analyzed using EZR (Saitama Medical Center, Jichi Medical University, Saitama, Japan) [22], a graphical user interface for R (The R Foundation for Statistical Computing, Vienna, Austria). Comparisons between the two groups were performed using the Mann–Whitney's *U* test. Multiple comparison procedures were conducted using the Kruskal–Wallis test. Differences were considered to be statistically significant at *p*-values <0.05.

## Data Availability

The data that support the findings of this study are available from the corresponding author upon reasonable request.

## Nomenclature

NRBC: nucleated red blood cell
HO-1: heme oxygenase 1
CB: umbilical cord blood
LPS: lipopolysaccharide
IL-10: interleukin 10
TNF-*α*: tumor necrosis factor-*α*
EB: erythroblast
ROS: reactive oxygen species
mAb: monoclonal antibody
RBC: red blood cells
HP: haptoglobin
PCR: polymerase chain reaction.

## Supporting Information

Supporting Information Table S1. Cord blood samples used in each experiment. Figure S1. Isolation strategy of cord blood derived NRBCs and monocytes. (A) CB mononuclear cells from fresh cord blood were separated to CD45+ cells and CD45− cells. CD14+ CD16− classical monocytes were isolated from CD45+ cells using flow cytometry. CD45− NRBCs were purified by positive selection using FITC-conjugated anti-CD36 mAbs. After separating CD45+ CD61+ from the CB mononuclear cells, using anti-CD45 mAb and anti-CD61 mAb, CD45− NRBCs were purified by positive selection using FITC-conjugated anti-CD36 mAb. (B) After separating CD45− CD61− from the CB mononuclear cells, using anti-CD45 mAb and anti-CD61 mAb, CD14+ CD16− classical monocytes were isolated from CD45+ cells with FITC-conjugated anti-CD14 mAb, PerCPcy5.5-conjugated anti-CD16 mAb and PI using flow cytometry. Abbreviations: CB, umbilical cord blood; NRBC, nucleated red blood cell; CBMC, CB mononuclear cell; FITC, fluorescein isothiocyanate; mAb, monoclonal antibody; PerCPcy5.5, peridinin chlorophyll protein-cyanine5.5; PI, propidium iodide. Figure S2. Flow cytometry gating method used to confirm purity of isolated cord blood-derived NRBCs and monocytes. (A) The purity of CD14+ CD16− monocytes. CD14+ CD16− classical monocytes were stained with FITC-conjugated anti-CD14 mAb, PerCPcy5.5-conjugated anti-CD16 mAb and PI and analyzed through flow cytometry. The purity of monocytes was analyzed using a flow cytometer. (B) The purity of CD45− NRBCs. The CD45− NRBCs were stained with PI to identify dead cells. The purity of NRBCs was analyzed by a flow cytometer after staining. FITC-conjugated anti- CD36 mAb, APC-conjugated anti-CD71 mAb, APC-Cyanine7-conjugated anti-CD235a mAb, PE-Cyanine7-conjugated anti-CD45 mAb. Abbreviations: CB, umbilical cord blood; NRBC, nucleated red blood cell; FITC, fluorescein isothiocyanate; mAb, monoclonal antibody; PerCPcy5.5, peridinin chlorophyll protein-cyanine5.5; PI, propidium iodide; APC, allophycocyanin; PE, phycoerytrin.
