# Supplementary material for: Nucleated Red Blood Cells Secrete Haptoglobin to Induce Immunosuppressive Function in Monocytes
Source: J Immunol Res. 2025 Feb 20;2025:8085784. doi: 10.1155/jimr/8085784 (PMC11867727; doi:10.1155/jimr/8085784)
Supplement: Supporting Information — Table S1. Cord blood samples used in each experiment. Figure S1. Isolation strategy of cord blood derived NRBCs and monocytes. (A) CB mononuclear cells from fresh cord blood were separated to CD45+ cells and CD45− cells. CD14+ CD16− classical monocytes were isolated from CD45+ cells using flow cytometry. CD45− NRBCs were purified by positive selection using FITC-conjugated anti-CD36 mAbs. After separating CD45+ CD61+ from the CB mononuclear cells, using anti-CD45 mAb and anti-CD61 mAb, CD45− NRBCs were purified by positive selection using FITC-conjugated anti-CD36 mAb. (B) After separating CD45− CD61− from the CB mononuclear cells, using anti-CD45 mAb and anti-CD61 mAb, CD14+ CD16− classical monocytes were isolated from CD45+ cells with FITC-conjugated anti-CD14 mAb, PerCPcy5.5-conjugated anti-CD16 mAb and PI using flow cytometry. Abbreviations: CB, umbilical cord blood; NRBC, nucleated red blood cell; CBMC, CB mononuclear cell; FITC, fluorescein isothiocyanate; mAb, monoclonal antibody; PerCPcy5.5, peridinin chlorophyll protein-cyanine5.5; PI, propidium iodide. Figure S2. Flow cytometry gating method used to confirm purity of isolated cord blood-derived NRBCs and monocytes. (A) The purity of CD14+ CD16− monocytes. CD14+ CD16− classical monocytes were stained with FITC-conjugated anti-CD14 mAb, PerCPcy5.5-conjugated anti-CD16 mAb and PI and analyzed through flow cytometry. The purity of monocytes was analyzed using a flow cytometer. (B) The purity of CD45− NRBCs. The CD45− NRBCs were stained with PI to identify dead cells. The purity of NRBCs was analyzed by a flow cytometer after staining. FITC-conjugated anti- CD36 mAb, APC-conjugated anti-CD71 mAb, APC-Cyanine7-conjugated anti-CD235a mAb, PE-Cyanine7-conjugated anti-CD45 mAb. Abbreviations: CB, umbilical cord blood; NRBC, nucleated red blood cell; FITC, fluorescein isothiocyanate; mAb, monoclonal antibody; PerCPcy5.5, peridinin chlorophyll protein-cyanine5.5; PI, propidium iodide; APC, allophycocyanin; PE, phycoe [file 8085784.f1.docx]

**Supplemental data contents**

Supplemental Table. Data collection. Cord blood samples used in each experiment

Supplemental Figure 1. Isolation strategy of cord blood derived NRBCs and Monocytes.

Supplemental Figure 2. Flow cytometry gating method used to confirm purity of isolated cord blood-derived NRBCs and monocytes

Supplemental table. Cord blood samples used in each experiment

|  | Identification number of the cord blood sample | | | | | | | | | | | | | | | | | | | | | |
| --- | --- | --- | --- | --- | --- | --- | --- | --- | --- | --- | --- | --- | --- | --- | --- | --- | --- | --- | --- | --- | --- | --- |
|  | 1 | 2 | 3 | 4 | 5 | 6 | 7 | 8 | 9 | 10 | 11 | 12 | 13 | 14 | 15 | 16 | 17 | 18 | 19 | 20 | 21 | 22 |
| Figure 1A and 1C | ○ | ○ | ○ | ○ | ○ | ○ | ○ |  |  |  |  |  |  |  |  |  |  |  |  |  |  |  |
| Figure 1B and 1D |  | ○ |  |  |  |  |  | ○ | ○ | ○ | ○ | ○ |  |  |  |  |  |  |  |  |  |  |
| Figure 2A |  |  |  |  |  |  |  |  |  |  |  |  | ○ | ○ | ○ | ○ | ○ |  |  |  |  |  |
| Figure 2B |  |  |  |  |  |  |  |  |  |  |  |  |  |  |  |  |  | ○ | ○ | ○ | ○ | ○ |


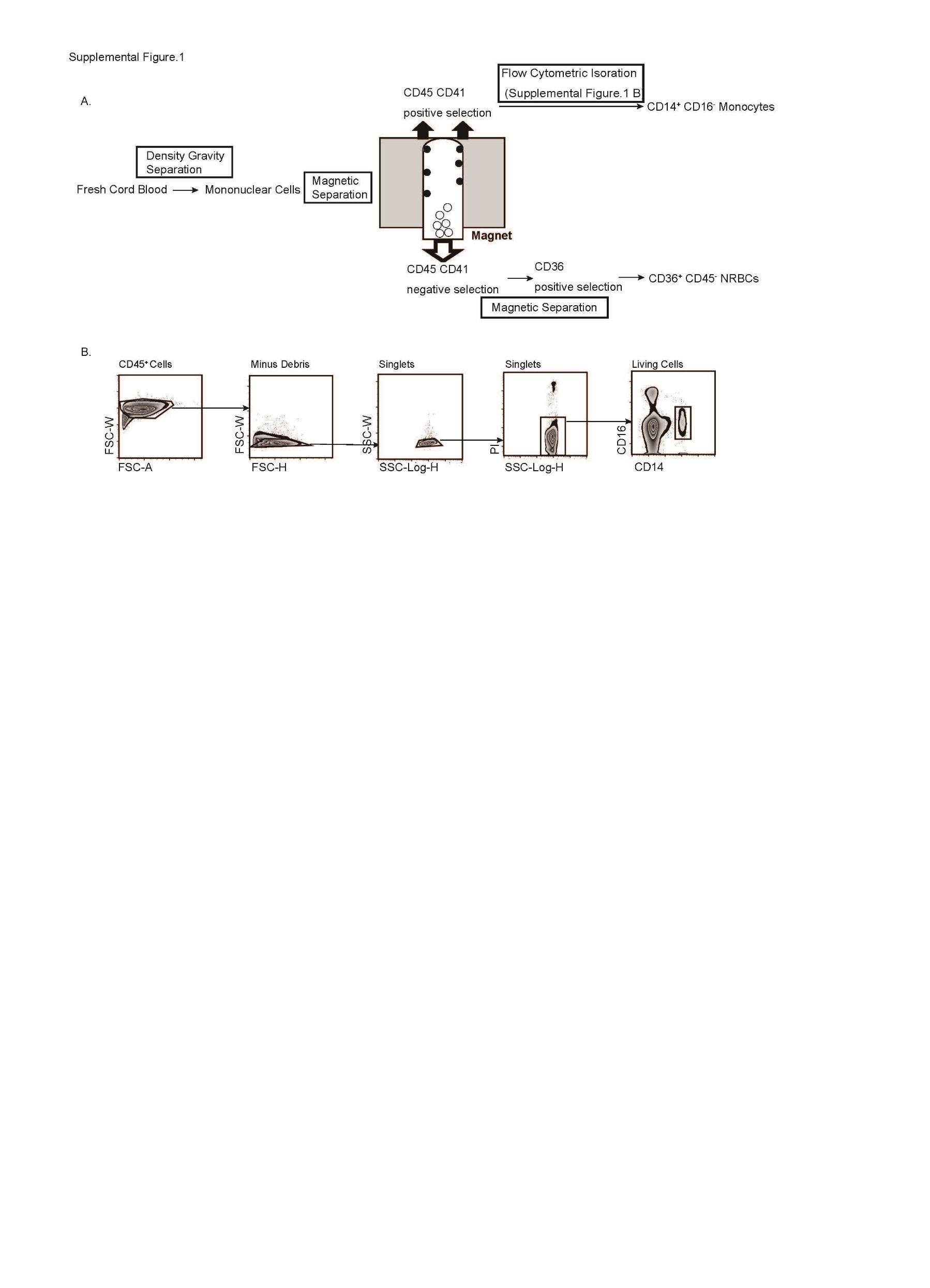


**Supplemental Figure 1. Isolation strategy of cord blood derived NRBCs and Monocytes.**

(A) CB mononuclear cells from fresh cord blood were separated to CD45+ cells and CD45- cells. CD14+CD16- classical monocytes were isolated from CD45+ cells using flow cytometry. CD45-NRBCs were purified by positive selection using FITC-conjugated anti-CD36 mAbs. After separating CD45+ CD61+ from the CB mononuclear cells, using anti-CD45 mAb and anti-CD61 mAb, CD45-NRBCs were purified by positive selection using FITC-conjugated anti-CD36 mAb. (B) After separating CD45- CD61- from the CB mononuclear cells, using anti-CD45mAb and anti-CD61 mAb, CD14+CD16- classical monocytes were isolated from CD45+ cells with FITC-conjugated anti-CD14 mAb, PerCPcy5.5-conjugated anti-CD16 mAb and PI using flow cytometry. Abbreviations: CB, umbilical cord blood; NRBC, nucleated red blood cell; CBMC, CB mononuclear cell; FITC, fluoresceinisothiocyanate; mAb, monoclonal antibody; PerCPcy5.5, peridinine chlorophyll protein-cyanine5.5; PI, propidium iodide.


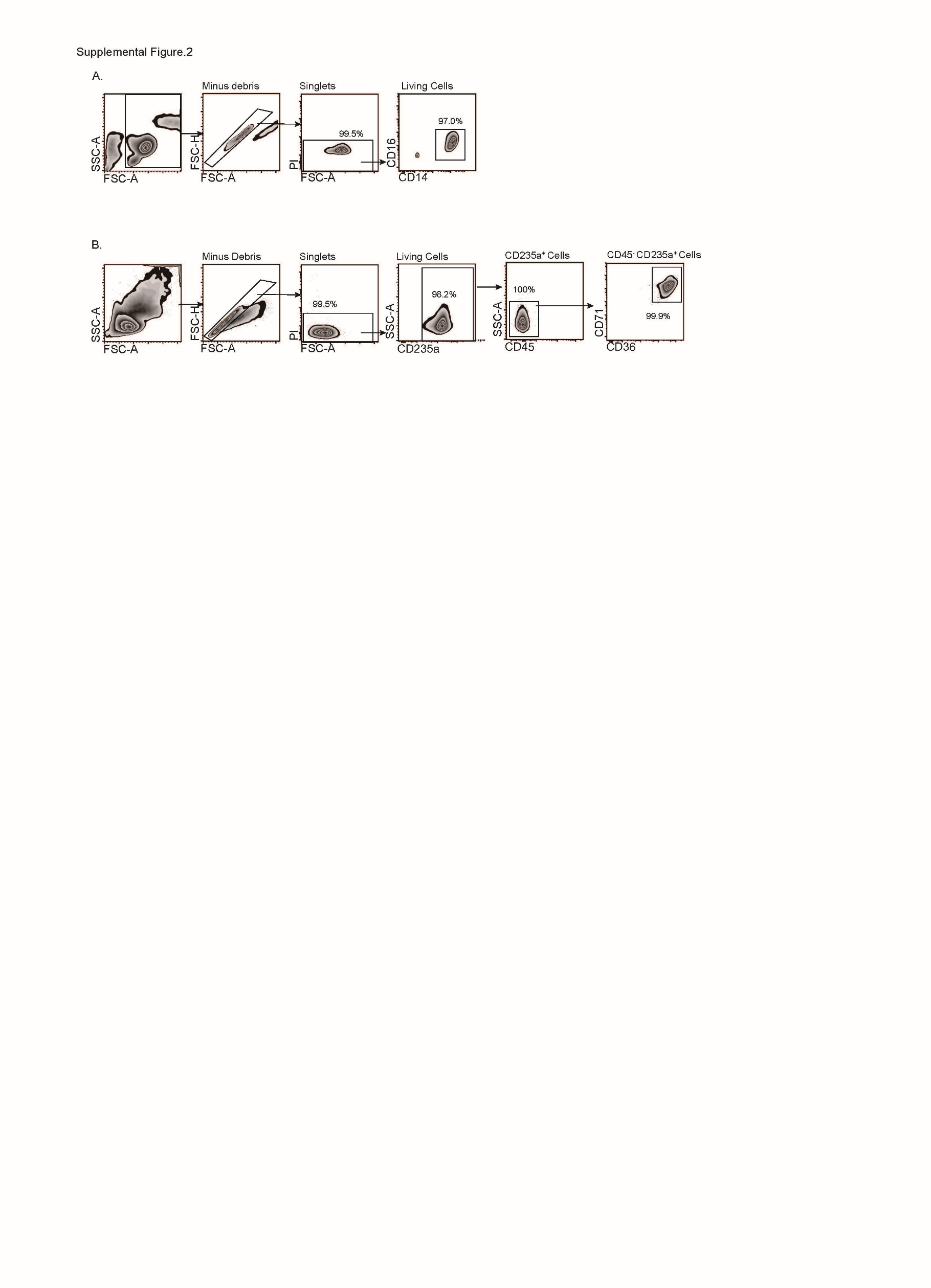


**Supplemental Figure 2. Flow cytometry gating method used to confirm purity of isolated cord blood-derived NRBCs and monocytes.**

(A) The purity of CD14+ CD16- monocytes. CD14+CD16- classical monocytes were stained with FITC-conjugated anti-CD14 mAb, PerCPcy5.5-conjugated anti-CD16 mAb and PI and analyzed through flow cytometry. The purity of monocytes was analyzed using a flow cytometer.

(B) The purity of CD45- NRBCs. The CD45-NRBCs were stained with PI to identify dead cells. The purity of NRBCs was analyzed by a flow cytometer after staining. FITC-conjugated anti- CD36 mAb, APC-conjugated anti-CD71 mAb, APC-Cyanine7-conjugated anti-CD235a mAb, PE-Cyanine7-conjugated anti-CD45 mAb. Abbreviations: CB, umbilical cord blood; NRBC, nucleated red blood cell; FITC, fluoresceinisothiocyanate; mAb, monoclonal antibody; PerCPcy5.5, peridinine chlorophyll protein-cyanine5.5; PI, propidium iodide; APC, allophycocyanin; PE, phycoerytrin.
